# Multi-omics data integration and modeling unravels new mechanisms for pancreatic cancer and improves prognostic prediction

**DOI:** 10.1038/s41698-022-00299-z

**Published:** 2022-08-17

**Authors:** Nicolas A. Fraunhoffer, Analía Meilerman Abuelafia, Martin Bigonnet, Odile Gayet, Julie Roques, Remy Nicolle, Gwen Lomberk, Raul Urrutia, Nelson Dusetti, Juan Iovanna

**Affiliations:** 1 Centre de Recherche en Cancérologie de Marseille (CRCM), INSERM U1068, CNRS UMR 7258, Parc Scientifique et Technologique de Luminy, Aix‐Marseille Université and Institut Paoli‐Calmettes, Marseille, France; 2 Universidad de Buenos Aires, Consejo Nacional de investigaciones Científicas y Técnicas. Centro de Estudios Farmacológicos y Botánicos (CEFYBO). Facultad de Medicina, Buenos Aires, Argentina; 3 Universidad de Buenos Aires, Facultad de Medicina, Departamento de Microbiología, Parasitología e Inmunología, Buenos Aires, Argentina; 4 Tumour Identity Card Program (CIT), French League Against Cancer, Paris, France; 5 Genomics and Precision Medicine Center (GSPMC), Medical College of Wisconsin, Milwaukee, WI USA; 6 Division of Research, Department of Surgery, Medical College of Wisconsin, Milwaukee, WI Center, Medical College of Wisconsin, Milwaukee, WI USA

**Keywords:** Predictive markers, Tumour heterogeneity

## Abstract

Pancreatic ductal adenocarcinoma (PDAC), has recently been found to be a heterogeneous disease, although the extension of its diversity remains to be fully understood. Here, we harmonize transcriptomic profiles derived from both PDAC epithelial and microenvironment cells to develop a Master Regulators (MR)-Gradient model that allows important inferences on transcriptional networks, epigenomic states, and metabolomics pathways that underlies this disease heterogeneity. This gradient model was generated by applying a blind source separation based on independent components analysis and robust principal component analyses (RPCA), following regulatory network inference. The result of these analyses reveals that PDAC prognosis strongly associates with the tumor epithelial cell phenotype and the immunological component. These studies were complemented by integration of methylome and metabolome datasets generated from patient-derived xenograft (PDX), together experimental measurements of metabolites, immunofluorescence microscopy, and western blot. At the metabolic level, PDAC favorable phenotype showed a positive correlation with enzymes implicated in complex lipid biosynthesis. In contrast, the unfavorable phenotype displayed an augmented OXPHOS independent metabolism centered on the Warburg effect and glutaminolysis. Epigenetically, we find that a global hypermethylation profile associates with the worst prognosis. Lastly, we report that, two antagonistic histone code writers, SUV39H1/SUV39H2 (H3K9Me3) and KAT2B (H3K9Ac) were identified key deregulated pathways in PDAC. Our analysis suggests that the PDAC phenotype, as it relates to prognosis, is determined by a complex interaction of transcriptomic, epigenomic, and metabolic features. Furthermore, we demonstrated that PDAC prognosis could be modulated through epigenetics.

## Introduction

Pancreatic ductal adenocarcinoma (PDAC) is one of the most aggressive tumors with a five-year survival rate between 3% and 30% depending on the diagnosis time, being the patients with distal metastasis those with the poorest prognoses. Based on its increasing incidence world-wide, PDAC is expected to become the second cause of cancer death by 2030^1–3^. The hallmarks that define PDAC prognosis and treatment response are determined by the progression and crosstalk of the tumor cell and its microenvironment compartments^4–6^. Specifically, the epithelial tumor cells have been extensively typified at a molecular level to capture patients’ outcomes^7–10^. Many current studies associate clinical findings primarily based on the classical/basal-like classifiers. Recently, however, our laboratory demonstrated the co-existence of multiple epithelial tumor cell subtypes into the same tumor^11^. Thus, we generated a PDAC distribution based on the histology and termed PDAC Molecular Gradient (PAMG)^12^ which was centered in the tumor epithelial cell.

Based primarily on epithelial cell characteristics, we and others have shown that a set of central transcriptional factors (TFs) defines the tumor cell phenotype. The classical subtype is characterized by ductal cell and germline linage TFs, such as PDX1, HNF4A, HNF1A, and GATA6, whereas the basal-like subtype is modulated by SNAI2, SIX1/4, and TP63^7,9,10,13–16^. Additional TFs are induced depending on intra-tumoral and extra-tumoral factors such as hypoxia and epithelial-mesenchymal transition (EMT) related proteins which contribute to tumor aggressiveness^17,18^. Therefore, the global PDAC phenotype is highly determined by a core of of transcription factors and epigenetic regulator proteins, which dynamics defines the patients’ outcome. Interestingly, this extensive characterization of PDAC TFs has been performed on the tumoral epithelial cell only, limiting the understanding of this regulatory network in the microenvironment. In the current study, we describe a PDAC prognosis model that incorporates data derived from key TFs, which act as master regulators (MR) of tumor cells and its microenvironment. We termed this new continuous stratification method as MR-Gradient. MR-Gradient combines both data-driven and experimentally derived inferences from transcriptional networks and their link with epigenomics and metabolomic profile. Analyses from this new modeling reveal three antagonistic writer enzymes, SUV39H1-SUV39H2 (H3K9Me3) and KAT2B (H3K9Ac), as novel pathways associated with PDAC heterogeneity. We also demonstrate that lipid metabolism not only plays a critical role in PDAC pathobiology but also associates with defined prognosis. The novelty and importance of these findings for a better understanding the pathobiology and management of this dismal malignant disease is discussed.

## Results

### Deconvoluted transcriptome-based stratification through both the transformed cells and microenvironment components improves prognostic prediction of PDAC

**Fig. 1:**
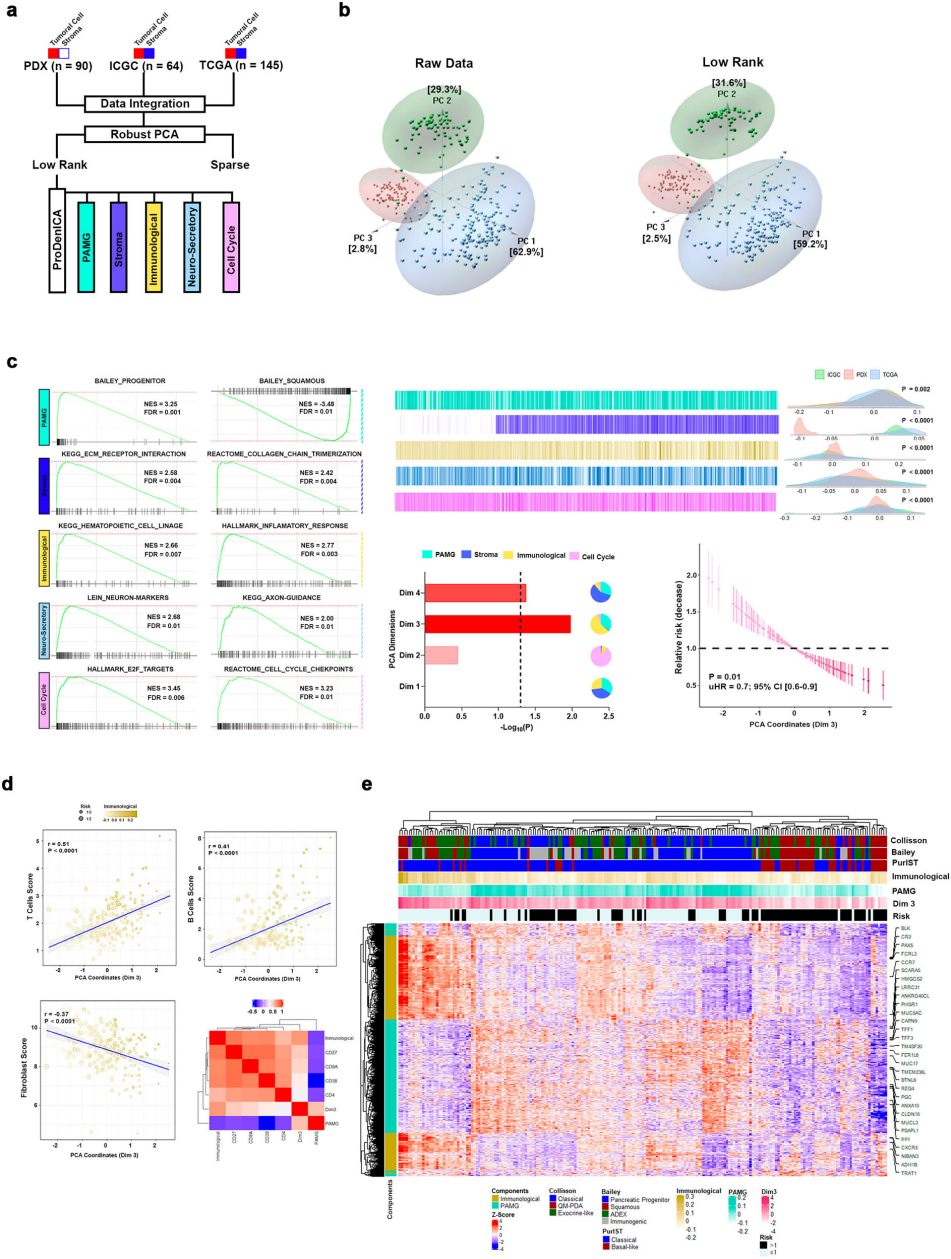
PDAC biological relevant components determination. **a** Low-Rank ICA (LRICA) methodology was applied on the integrated PDX, TCGA-PAAD, and ICGC-PACA-AU Seq datasets to establish the tumor phenotype gradients. **b** Comparing the original integrated expression matrix with the Low-Rank matrix applying principal component analysis (PCA). **c** Five PDAC biological relevant components were detected after LRICA, applying as the selection criteria, the kurtosis, and the biological enrichment through GSEA. Also, PCA was applied to evaluate the component contribution to the patient’s prognosis. Dimension 3 (Dim 3) showed the highest association with the prognosis (uHR = 0.76, 95% CI [0.62–0.94]; P = 0.01). This dimension was explained mainly by the PAMG (35.8%) and Immunological (57.2%) components. **d** T (*r* = 0.51; *P* < 0.0001) and B (*r* = 0.41; P < 0.0001) cells scores after stroma deconvolution of bulk RNA displayed a positive correlation with the Dim 3, whereas the fibroblast (*r* = −0.37; *P* < 0.0001) score was negative. This observation was complemented by correlation of the T and B cell markers. Specifically, CD8A and CD27 displayed the highest coefficients. **e** Heatmap of the high contributive gene of PAMG and Immunological components for TCGA-PAAD, and ICGC-PACA-AU Seq datasets.

We generated a discovery cohort of patient data that could capture PDAC heterogeneity by numerically harmonizing and mining data derived from three RNA expression datasets, our own experimental data from 90 patient-derived xenografts (PDXs) and two available from public domains (TCGA-PAAD and ICGC-PACA-AU Seq). Similar normalization strategies have been previously used to draw inferences of biological significance when working with expression asymmetry across datasets^7,8^. Therefore, this data appropriately represents molecular events tightly associated with the pathobiology of PDAC and serves as a useful baseline to build mechanistically oriented prediction models for prognosis and therapies. Toward this end, we applied Low-Rank ICA (LRICA) to the analyses of the discovery cohort, considering that key aspects of PDAC pathobiology are intrinsically low-dimensional in nature^19^. We also used robust principal component analyses (RPCA) to decompose our expression matrix into a low-rank (L) and a sparse (S) one (Fig. 1a). This approach allowed us to separate the underlining biology (L) and noise (S) of the tumor cell population, without modifying the overall structure of expression matrices and proximity among datasets (Fig. 1b). We subsequently applied ProDenICA with the goal of increasing the information that can be gained from the L matrix. In this approach, the selection and directionality of each component was determined by excess kurtosis (to evaluate normal value distributions) and biological relevance measured by GSEA, which resulted in five components (Fig. 1c; Supplementary Table 1). Supporting the validation of this approach, we found a component termed PAMG which capture the tumor epithelial cell phenotype, which displays strong polarization to the progenitor (NES = 3.25; FDR = 0.001; Fig. 1c) and squamous phenotype (NES = −3.48; FDR = 0.01; Fig. 1c). In addition, this method differentiated two microenvironment-specific components, including one primarily defined by fibroblast-related features, such as MYCAF (NES = 2.26; FDR < 0.0001; Supplementary Fig. 1a) and ICAF (NES = 2.59; FDR < 0.0001; Supplementary Fig. 1a). The second was an immunological component, which captures information on hematopoietic lineages (NES = 2.06; FDR = 0.007; Fig. 1c) and inflammatory processes (NES = 2.77; FDR = 0.003; Fig. 1c). Lastly, we also identified a neuro-secretory and a cell cycle component. Thus, this approach yields more appropriately weighted information on the type of biological information that resides within the mathematical structure of the data and contributes to the bioinformatics-based modeling of pancreatic cancer-associated processes.

Next, we determined the association of the components defined above with patient overall survival (OS). For this purpose, we used TCGA-PAAD and ICGC-PACA-AU Seq, as the discovery cohort for downstream analysis. At this point, we excluded our human xenograft dataset to avoid any bias related to lack of microenvironment compartments. Kaplan–Meier survival analysis revealed that patient OS highly correlated to PAMG (*P* = 0.004), stroma (*P* = 0.01), immunological (*P* = 0.004), and cell cycle (*P* = 0.03) components (Supplementary Fig. 1b). Unfavorable prognosis correlated with the activation of cell cycle and EMT pathways (Supplementary Fig. 1b; Supplementary Table 1). On the other hand, we found a high correlation of patients with a favorable prognosis and enrichment in lipid metabolism and immunological pathways (Supplementary Fig. 1b; Supplementary Table 1). Interestingly, the immunological-related pathways determined the prognosis profile within the stroma component (Supplementary Fig. 1b; Supplementary Table 1), highlighting the importance of including the microenvironment component as a prognostic marker.

To weight the contribution of the Kaplan–Meier analysis significant components on the prognosis, PCA followed by a Cox proportional-hazards model on the PCA coordinates were applied. This analysis indicated that both Dimension 3 (Dim 3; uHR = 0.76, 95% CI [0.62–0.94]; *P* = 0.01) and Dimension 4 (Dim 4; uHR = 0.8, 95% CI [0.65–0.99]; *P* = 0.04) significantly associated with the OS (Fig. 1c, Supplementary Fig. 1c), with Dim 3 as the strongest contributor. This observation was confirmed by multivariant cox regression analysis, where the Dim 3 remains significantly associated with the OS (mHR = 0.78, 95% CI [0.63–0.98]; *P* = 0.03; Supplementary Table 1). Dimension 3 was primarily represented by the immunological component with correlation coefficients of 0.68 (*P* < 0.0001; Fig. 1d). Moreover, additional deconvolution of stromal compartments suggested that favorable prognosis displayed positive correlation with T cells and B cells, while a negative one with fibroblast abundance (Fig. 1d; Supplementary Fig. 1d; Supplementary Table 1), an implication of both biological and medical relevance. Specifically, the cytotoxic cell marker, CD8A (*r* = 0.44; *P* < 0.0001; Supplementary Table 1), and two plasma cell markers, CD27 (*r* = 0.44; *P* < 0.0001; Supplementary Table 1) and CD38 (*r* = 0.30; *P* < 0.0001; Supplementary Table 1), positively correlated with Dim 3. Taken together, these results highlight the fact that PDAC prognosis is not determined by the tumor cell or the microenvironment in isolation, but rather through their combined contribution. Hence, by highlighting this interdependency, our data should contribute to better conceptualize the search and development of both markers and targeted drugs. Lastly, we reveal that the stratification of PDAC as a molecular gradient, according to PAMG, when enhanced by the contribution of the immunological components (Fig. 1e), significantly differentiates patient clinical outcomes.

### Transcriptional regulatory network analyses provide pathobiological information and yield useful molecular markers for patient stratification

**Fig. 2:**
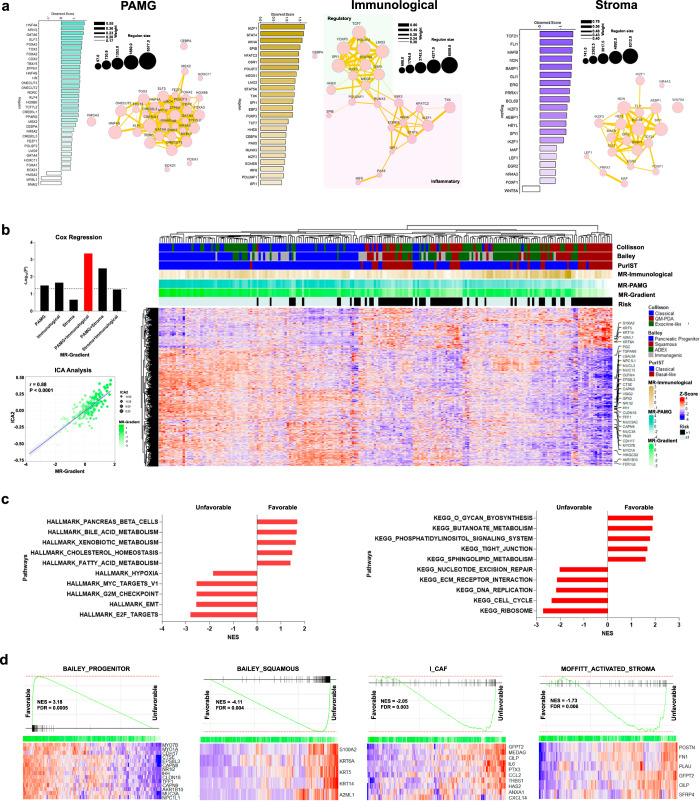
Transcriptional Master Regulator (MR) gradient and tumoral phenotype characterization. **a** Transcriptional network and enrichment score of key regulons after regulatory transcriptional network (RTN) analysis on PAMG, immunological, and stroma components. **b** Univariate Cox regression Log-Rank *P*-value representation applying the gradients of master regulators (MR) extracted from the RTN analysis for TCGA-PAAD, and ICGC-PACA-AU Seq datasets. Moreover, the global PDAC phenotype associated to the MR-Gradient (integration of PAMG and immunological MR) was extracted using ICA. ICA2 was detected (*r* = 0.88; *P* < 0.0001) and the high contributive genes were represented in a heatmap. **c** Gene-set enrichment analysis (GSEA) was applied on ICA2, detecting the pathways associated with a favorable and unfavorable prognosis. **d** MR-Gradient capture both tumor epithelial cell and microenvironment features, previously associated with the prognosis, such as BAILEY_PROGENITOR (NES = 3.18; FDR = 0.0005), BAILEY_SQUAMOUS (NES = −4.11; FDR = 0.004), I_CAF (NES = −2.05; FDR = 0.003), and MOFFITT_ACTIVATED_STROMA (NES = −1.73; FDR = 0.006).

We performed transcriptional regulatory networks analyses on the same two public datasets used above to unravel key upstream modulators governing each of the components that contribute to prognostic predictions. We focused our analysis on connected transcriptional factors (TFs) with high contribution to the LRICA components displaying significant associations with OS, namely PAMG as well as the immunological and stroma components. Initially, we constructed a regulatory transcriptional network (RTN) for each component, using the ARACNe algorithm^20,21^ for the TFs identified through GO annotation (GO:0001067; Regulatory region nucleic acid binding). Using this approach, we detected a total of 113 TFs, representing 121 regulons with a range of molecular interactions between 16 and 5312 events. Subsequently, we identified the master regulators (MR) within the TF set by testing for the enrichment of each regulon associated with the specific component (Supplementary Table 1). Accordingly, we detected 54 MR with an absolute enrichment score > 1 (Supplementary Table 1). PAMG displays a compact interaction network (Jaccard index ≥ 0.17; Fig. 2a), driven mainly by progenitor-related regulons (88%), in which HNF4A, NR1I2, and GATA6 showed the highest contribution. In addition, the squamous MR network was associated with SNAI2, MYBL1, and HMGA2, which are key regulators of EMT and cell cycle progression^17,22,23^. Moreover, we observed an immunological MR network polarized into modulatory and proinflammatory nodes (Jaccard index ≥ 0.30; Fig. 2a), characterized by TFs related to Treg-cell (FOXP3 and STAT5) and B/T-cell activation (IKZF1 and NFATC2). Lastly, the stroma component was characterized for pleiotropic TFs that represents multiple microenvironment cell types. However, enrichment in immunological-related regulators, such as MAFB, BCL6B, IKZF3, and SP1 was observed (Fig. 2a). Once establishing the transcriptional regulatory network for each prognosis-relevant component, we hypothesized that the MR could accurately infer patient prognosis capturing the cell global phenotype in unbiased way. To test the validity of this idea, we applied a Cox univariate proportional-hazards model to each MR to evaluate their predictive power. We found that HMGA2, SNAI2, GATA6, and ZFPM1 display the highest association with prognosis, independent of the cohort (Supplementary Fig. 2) used for our evaluation. However, to generate a consistent stratification that captured both, the epithelial and the microenvironment features, we built a unified gradient with the MR extracted from the transcriptional network analysis where each gradient was computed, weighting the gene expression (GE) with the enrichment score (ES) for each MR (i) and patient (j), followed by the scaled summation (see Eq. 1, Materials and methods). This analysis reveals a combined contribution from PAMG and immunological transcription factors outperformed the other components, even when combined, to estimate patient prognosis in the discovery cohort (uHR = 0.7, 95% CI [0.60–0.86]; *P* = 0.0005; Supplementary Fig. 2a), ICGC-Array (uHR = 0.69, 95% CI [0.53–0.89]; *P* = 0.006; Supplementary Fig. 2b), and Puleo (uHR = 0.74, 95% CI [0.63–0.87]; *P* = 0.0002; Supplementary Fig. 2c). We termed this new continuous stratification method as MR-Gradient. Notably, this MR-Gradient simplifies the prognosis estimation using a set of 40 MR that capture both, epithelial and microenvironment features. Then, we implemented the ICA JADE algorithm to the discovery cohort to unravel the global phenotype associated with the MR-Gradient. ICA2 showed a positive correlation with the MR-Gradient (*r* = 0.88; *P* < 0.0001; Supplementary Table 2), displaying a solid association between the CHOLESTEROL_HOMEOSTASIS (NES = 1.53; FDR = 0.03; Fig. 2c) and FATTY_ACID_METABOLISM (NES = 1.45; FDR = 0.02; Fig. 2c) pathways with a favorable prognosis, whereas an unfavorable phenotype correlated with upregulation of HYPOXIA (NES = −1.89; FDR = 0.01; Fig. 2c), EMT (NES = −2.61; FDR = 0.01; Fig. 2c) and CELL_CYCLE (NES = −2.41; FDR = 0.02; Fig. 2c) pathways. Lastly, we evaluated the value of this PDAC prognosis profile for capturing previously established subtypes. We found that ICA2 encompassed the key features that determine patient outcomes at both, the tumor cell and microenvironment levels, representing the progenitor/squamous spectrum and activated stroma, respectively (Fig. 2d). Together, these results demonstrate that a refined PDAC gradient-based in MR is a robust clinically actionable tool for patient stratification.

### Patient-derived xenografts (PDXs) recapitulate key PDAC prognosis-related features

**Fig. 3:**
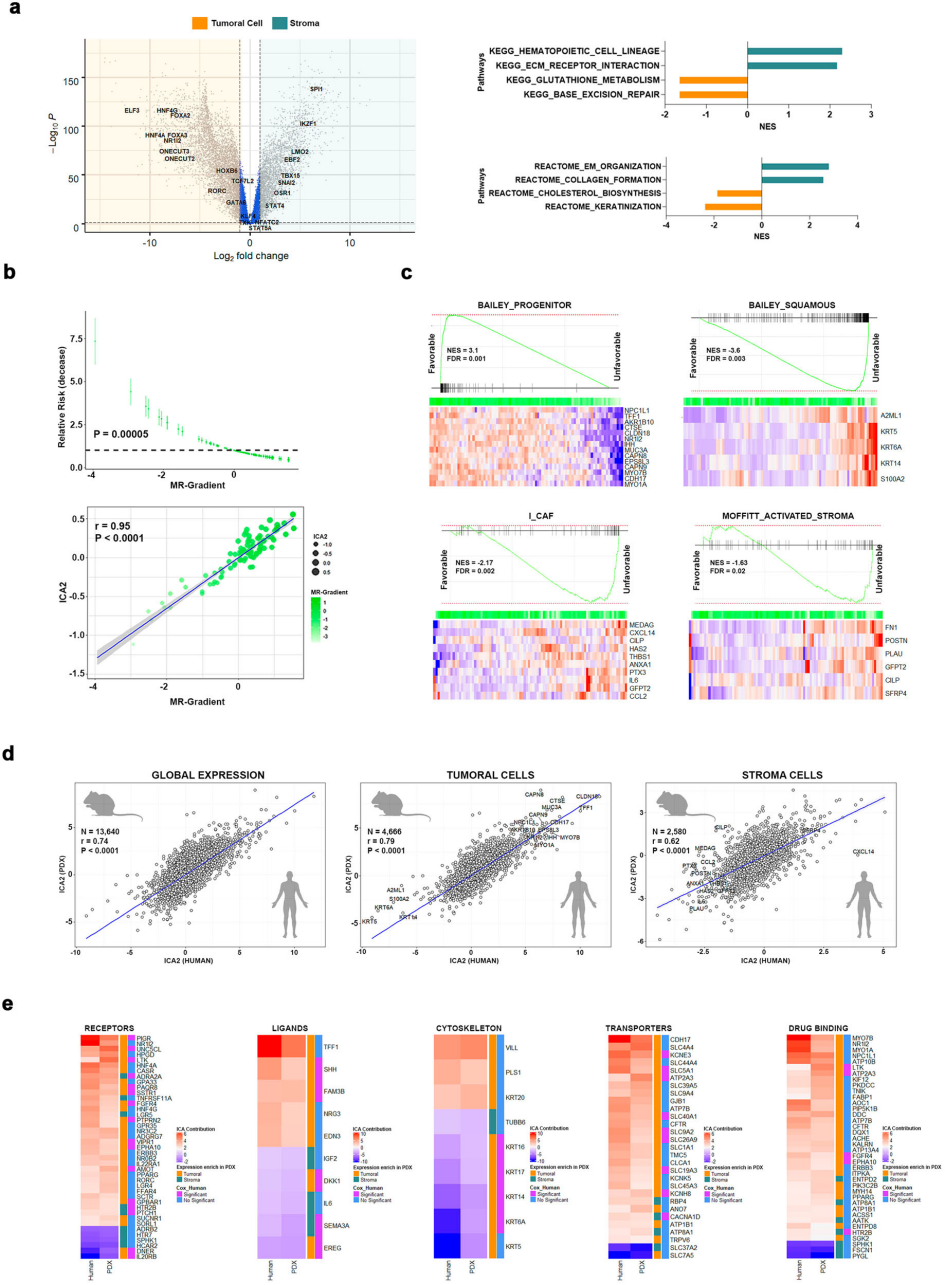
Patient-derived Xenografts recapitulate key determinants of PDAC prognosis. **a** Differential expression analysis was performed on the PDX tumor epithelial cell (human) and microenvironment (mice) matrices to evaluate the master regulators (MR) enrichment and pathways polarization. **b** Cox regression analysis confirmed the PDX transcriptional homology scoring significantly the patients following the MR-Gradient (uHR = 0.6, 95% CI [0.47–0.77]; *P* = 0.00005). In addition, we implemented ICA on the PDX expression matrix to identify the biological component that explains the MR-Gradient. **c** PDX ICA2 captured key pathways related with PDAC prognosis, including the tumor epithelial cell with the BAILEY_PROGENITOR (NES = 3.1; FDR = 0.0005), BAILEY_SQUAMOUS (NES = −3.6; FDR = 0.003) frame and microenvironment with I_CAF (NES = −2.17; FDR = 0.001), and MOFFITT_ACTIVATED_STROMA (NES = −1.63; FDR = 0.009). **d** Human and PDX ICA components displayed a high homology globally (*r* = 0.74; *P* < 0.0001) and both in tumor epithelial cell (*r* = 0.79; *P* < 0.0001) and microenvironment (*r* = 0.62; *P* < 0.0001) stratum, assigning equivalent weight to central PDAC biological markers. **e** Comparing gene contribution of human and PDX ICA components related with prognosis-related phenotype.

To expand our PDAC characterization at a multi-OMICs level, we used a set of 90 PDXs, which have demonstrated their utility as a tool to derive molecular signatures of significant medical relevance^4,5,12,24,25^. Since our PDX cohort has undergone data-driven deconvolution to distinctly represent the epithelia cell (human) and the microenvironment (mice), which may be contributed by the host, we performed differential expression analyses to validate the MR that characterize both compartments. MR represented within both PAMG and the immunological compartment displayed higher expression levels in the human tumor cell and murine microenvironment, respectively (Fig. 3a; Supplementary Table 2). GSEA revealed an enrichment of metabolic and epithelial cell differentiation pathways in the human fraction of the PDXs, as well as upregulation of hematopoietic and ECM pathways in the murine stromal compartment (Fig. 3a). Subsequently, we reconstituted the PDX expression matrix adding the human and mice compartment expression matrices to estimate the MR-Gradient and consequently weighed its predictive capability on the patient outcome. Noteworthy, univariate Cox regression analysis showed a significant positive correlation between the PDX-derived MR-Gradient and patient OS (uHR = 0.6, 95% CI [0.47–0.77]; *P* = 0.00005; Fig. 3b). In addition, we measured similarities in transcriptomics between our PDX and discovery cohorts to validate its use as a reliable representation of the defining prognostic features. Thus, we unraveled the PDX mixed matrix into latent biological spaces using the ICA JADE algorithm, which identified that ICA2 highly correlated with the MR-Gradient (*r* = 0.95; *P* < 0.0001; Fig. 3b). This component displayed the same phenotypic polarization observed in the discovery cohort (Supplementary Table 2), capturing the progenitor (NES = 3.1; FDR = 0.001; Fig. 3c) and squamous (NES = −3.6; FDR = 0.003; Fig. 3c) signatures, together with the microenvironment-derived poor prognosis profile for ICAF (NES = −2.16; FDR = 0.002; Fig. 3c) and activated stroma (NES = −1.63; FDR = 0.02; Fig. 3c). Furthermore, ICA2 from the discovery cohort and ICA2 from the PDX group revealed high correlation at both, the transformed cell (*r* = 0.74; *P* < 0.0001; Fig. 3d) and stroma (*r* = 0.62; *P* < 0.0001; Fig. 3d) levels, maintaining the directionality of gene contributions for key PDAC cellular features, such as for example cytoskeletal proteins and metabolic transporters among others (Fig. 3e). Altogether, these results validate the application of PDX-derived data into our analytical framework of a gradient in a manner that expands our inferences on PDAC prognosis.

### The CpG methylation profile contributes to defining the PDAC transcriptomic phenotype

**Fig. 4:**
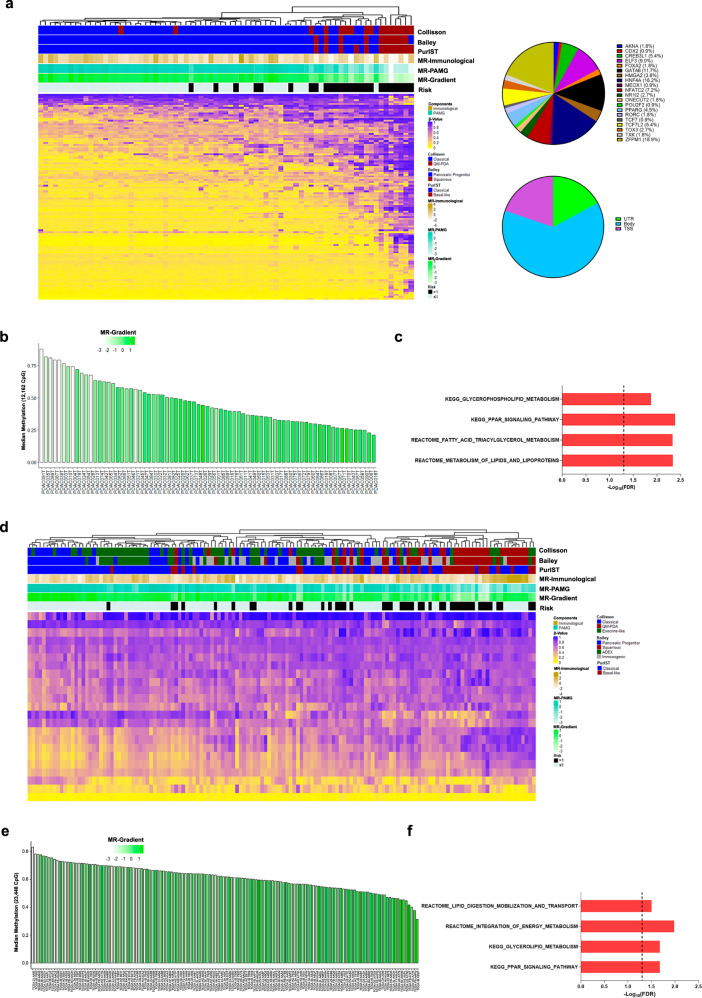
Association of the CpGs methylation profile with the MR-gradient. **a** The master regulators associated with a favorable prognosis showed a negative correlation between the methylation level and MR-Gradient. **b** Globally the unfavorable prognosis phenotype was associated with a hypermethylation profile of the ICA high contributive CpGs. **c** ICA high contributive CpGs were associated mainly with lipid metabolic pathways. **d** Master regulators related to a favorable prognosis displayed a hypermethylated profile in the unfavorable prognosis patients in the TCGA-PAAD cohort. **e** The global methylation level showed a negative correlation with the MR-Gradient in the TCGA-PAAD cohort. **f** ICA high contributive CpGs were associated with metabolic pathways in the TCGA-PAAD cohort.

Despite the central role of a well-known mutation landscape as the driver of the PDAC metaplastic phenotype, the lack of mutation diversity fails to explain tumor evolution and the basis for prognosis-related features^26^. Nevertheless, epigenetic plasticity contributes to PDAC heterogeneity^4,27^. Thus, we first analyzed DNA methylation levels of the MR. Broadly, key MR that determine the progenitor phenotype showed strong hypermethylation and associated with a poor prognosis, particularly ZFPM1, GATA6, and HNF4A (Fig. 4a). In addition, ICA was performed to capture the methylome profile related to patient outcome. A total of 12,162 significant (SD ≥ 3) CpG were selected into the component, and their methylation degree was analyzed as the median of the *β*-value per patient. Like the progenitor-related TR, we observed an increase in DNA methylation levels associated with a MR-Gradient decrease (Fig. 4b). Interestingly, this component was enriched for CpGs implicated in lipid metabolic pathways, including GLYCEROPHOSPHOLIPID METABOLISM (FDR = 0.01) and FATTY ACID TRIACYLGLYCEROL METABOLISM (FDR = 0.004). These results were confirmed on TCGA-PAAD cohort, where 23,448 CpGs displayed a high contribution in the selected component (Fig. 4d–f; Supplementary Table 3). Thus, the DNA methylation status of MR serves as a potential epigenetic mechanism that contributes to the PDAC prognosis-related phenotype.

### SUV39H1/2 and KAT2B are two antagonistic histone-based pathways that contribute to establishing the PDAC transcriptomic profile

**Fig. 5:**
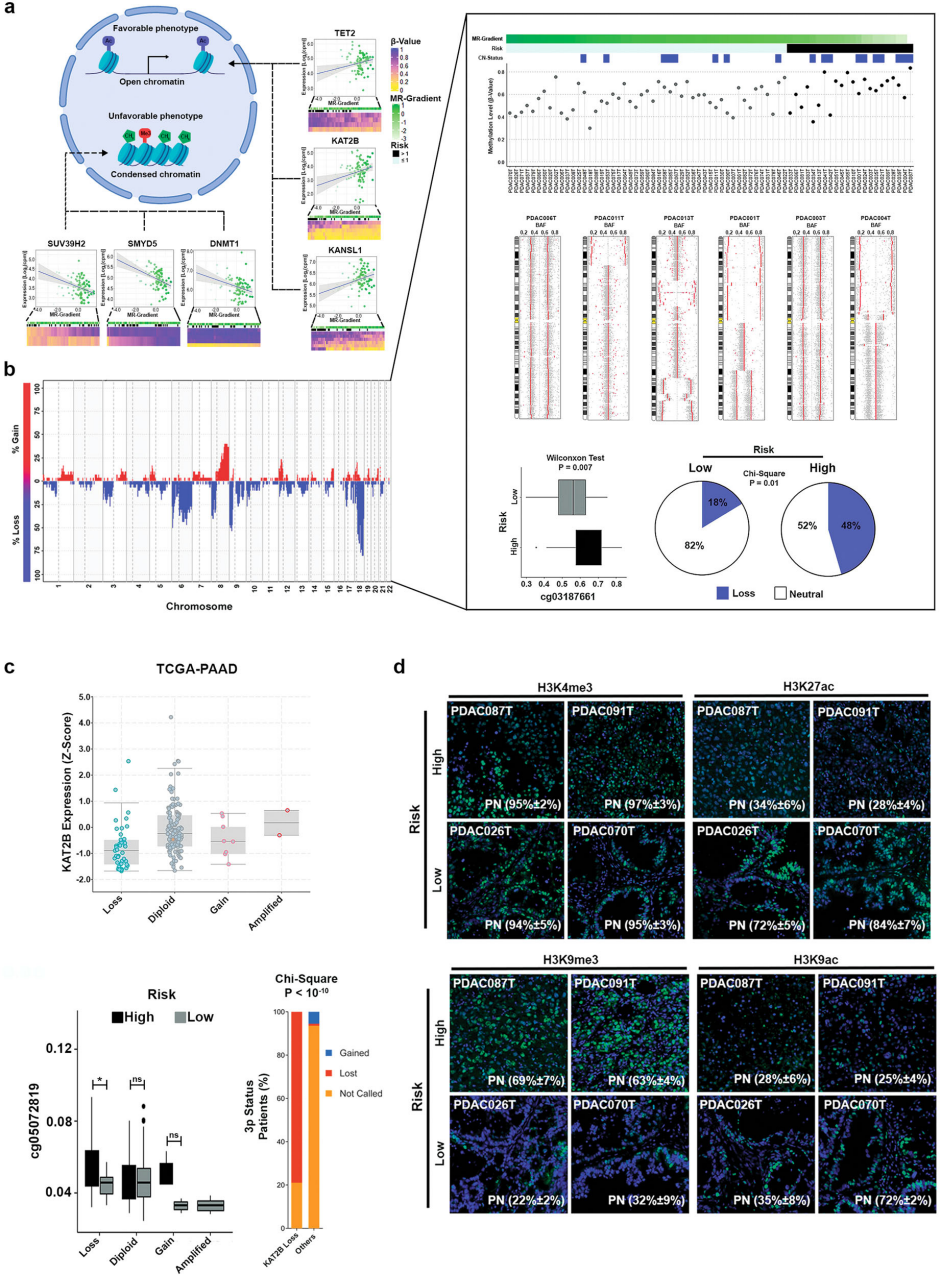
Identification of key epigenetic modifiers related with the PDAC prognosis phenotype. **a** One hundred forty-nine chromatin modifiers correlated with the MR-Gradient at transcriptional level. Specifically, SUV39H1/H2 and KAT2B, two antagonist writers of the H3K9 residue were identified correlating negatively and positively with the MR-Gradient, respectively. **b** KAT2B displayed a shallow deletion in the 25% of PDX which was associated with a high-risk phenotype (*P* = 0.01). Moreover, KAT2B expression levels were modulated by the balance between the shallow deletion and the methylation profile. **c** KAT2B shallow deletion was confirmed in the TCGA-PAAD cohort and was significantly associated with a hypermethylation in the high-risk patients and 3p status. Error bars indicate mean ± SD. *P < 0.05. **d** Immunofluorescence analysis of H3K9me3, H3K9ac, H3K4me3 and H3K27ac, confirmed the polarization of SUV39H1/H2 and KAT2B into the MR-Gradient.

Subsequently, we explored the correlation of multiple histone modifiers and readers, as additional epigenetic regulators, with our phenotype categories to infer mechanisms that regulate transcriptional outcomes. We found 149 proteins had significant correlation with the MR-Gradient (Supplementary Table 3). We identified SUV39H1 (*r* = −0.41; *P* < 0.0001; Fig. 5a), SUV39H2 (*r* = −0.39; *P* = 0.0001; Fig. 5a) and KAT2B (*r* = 0.31; *P* = 0.003; Fig. 5a), which are writers with a clear antagonistic role on the H3K9 residue, namely methylation for repression (SUV39H1/2) vs. acetylation with activation (KAT2B). Notably, in contrast to SUV39H1/2, we found that genomic deletion of KAT2B occurs in 25% of our cohort (Fig. 5b), a data confirmed by TCGA (Fig. 5c). Interestingly, this chromosomal loss displayed a balance with KAT2B promoter methylation, regulating its expression and consequently the prognostic phenotype (Fig. 5c). Moreover, KAT2B downregulation is an important characteristic of the squamous phenotype (Fig. 5c; Supplementary Table 3). We therefore quantified the specific epigenetic marks, namely H3K9me3 for SUV39H1 and SUV39H2 and H3K9ac for KAT2B, in a set of PDX samples that represents the MR-Gradient extremes. In addition, we complement the histone mark analysis using as reference two well-known activation marks, H3K4me3 and H3K27ac, which have displayed a general and a polarized expression pattern associated with PDAC phenotype, respectively. H3K9me3 and H3K9ac displayed opposite patterns, in which the trimethylation mark dominated the epigenetic landscape of high-risk patients with approximately 60% of positive nuclei (Fig. 5d). Conversely, K9 acetylation was prevalent in the group with favorable outcomes, along with high levels of H3K27ac (Fig. 5d). We used the H3K4me3 staining as a control mark since it displays a homogeneous expression level independently of the phenotype (Fig. 5d).

**Fig. 6:**
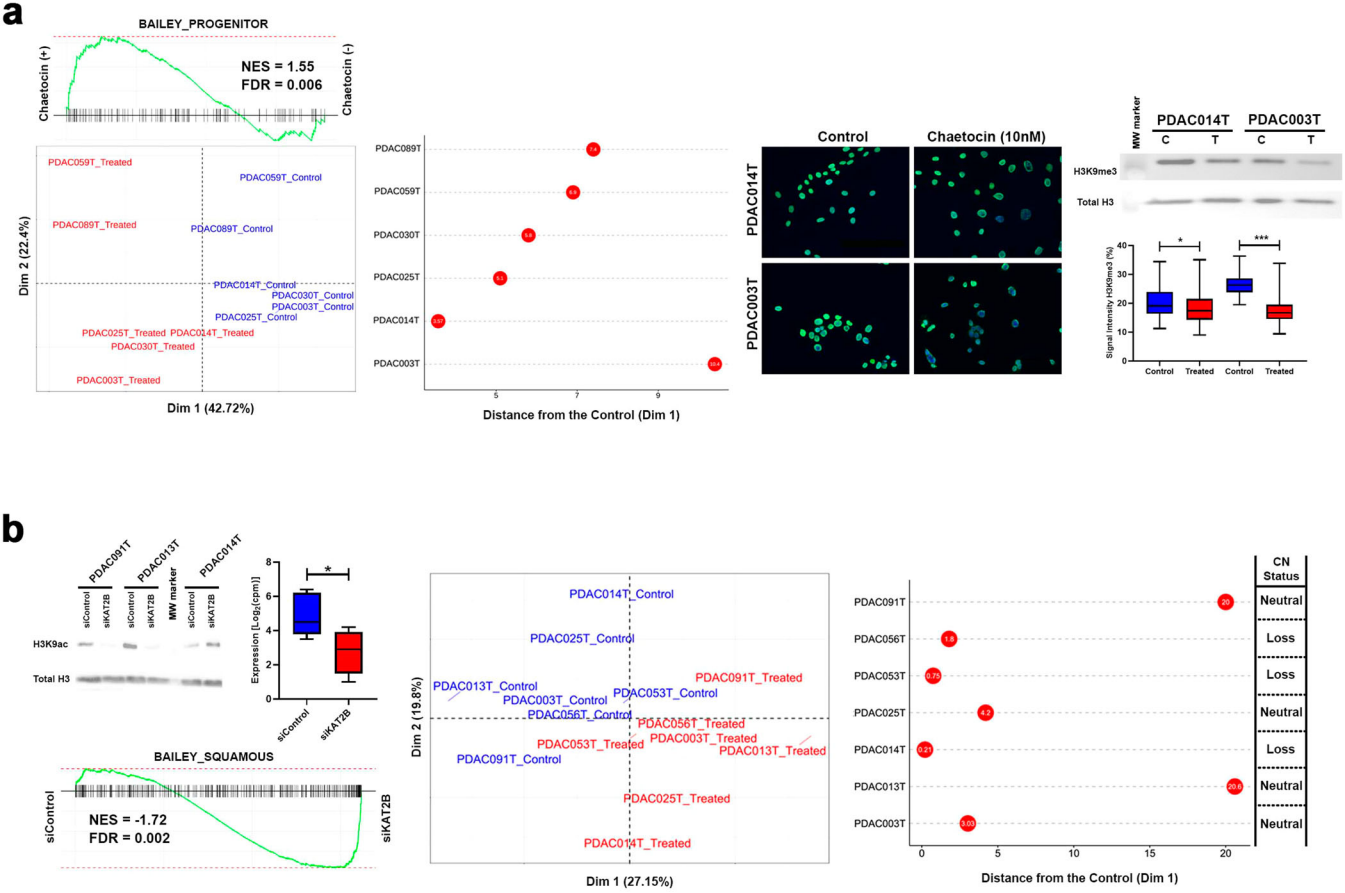
Modulation of SUV39H1/H2 and KAT2B determine the PDX-Derived Primary Cell Culture (PDPCC) phenotype. **a** Chemical modulation of SUV39H1/H2 through chaetocin (10 nM) for 72 h lead in the activation of Progenitor-related genes. Error bars indicate mean ± SD. *P < 0.05, **P < 0.01, ***P < 0.001. **b** KAT2B silencing using siRNA promotes the squamous program and was associated with the PDPCC deletion status. Error bars indicate mean ± SD. *P < 0.05.

Functional validation was performed through inhibiting or depleting SUV39H1/2 or KAT2B respectively. We used chaetocin, a mycotoxin with specific action on SUV39H1/2, at 10 nM on 6 PDX-derived Primary Cell Culture (PDPCC), which reduced H3K9me3 levels (Fig. 6a; Supplementary Fig. 3a) and was accompanied by the upregulation of progenitor-related genes (Fig. 6a; Supplementary Table 3). Levels of H3K9ac were reduced through using a set of specific KAT2B siRNAs (Fig. 6b; Supplementary Fig. 3b). KAT2B downregulation resulted in a squamous-like phenotype which is more obvious in PDPCC with neutral CNV as presented in Fig. 6b.

Combined, these results demonstrate a strong association between the transcriptional networks, epigenomic regulators, and patient outcome with the methylation level and H3K9 status as a determinant of this phenomenon. This new data also expands our previous observations on epigenomic landscapes of PDAC^23^ by linking two antagonistic writers, SUV39H1/2, and KAT2B, to PDAC pathobiology.

### Data integration links MR-Gradient to metabolomic functions that underlie the prognosis-related phenotypes

**Fig. 7:**
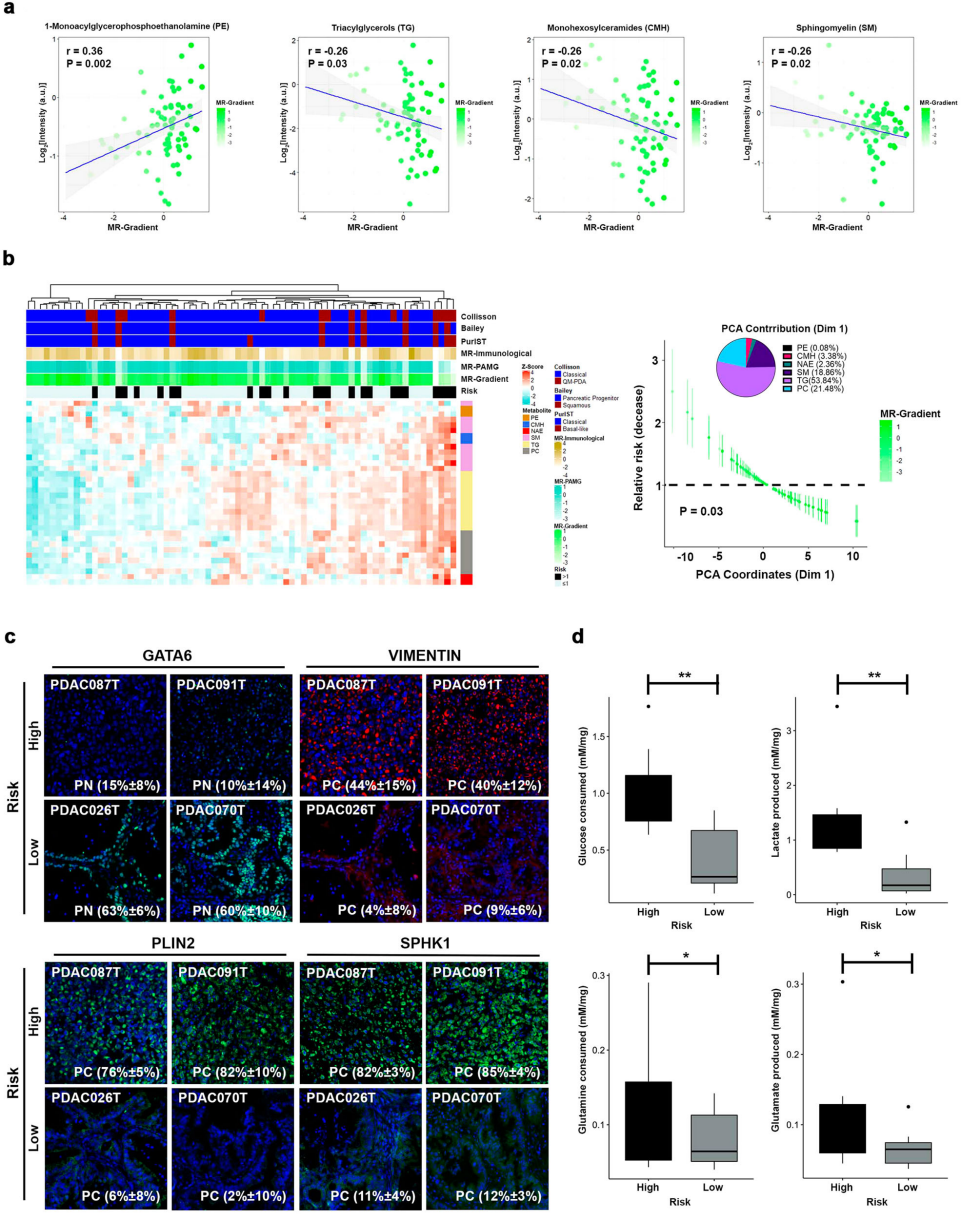
MR-Gradient capture key metabolic features associated with PDAC prognosis. **a** Correlation between the lipid subfamilies and MR-Gradient, identified the glycerophospholipids (*r* = 0.36; *P* = 0.002), sphingolipids metabolites (*r* = −0.26; *P* = 0.02), and triacyclglycerols (*r* = −0.26; *P* = 0.03) as determinant of PDAC prognosis. **b** Principal component analysis (PCA) on the high correlated metabolites from the prognosis-related lipid subfamilies, showed that the dimension 1 (Dim 1; uHR = 0.9, 95% CI [1.01–1.18]; *P* = 0.03) explains the prognosis and confirms the lipid metabolism as key prognosis determinant through the glycerophospholipids, sphingolipids metabolites, and triacyclglycerols. **c** GATA6 and VIMENTIN were associated with the MR-Gradient. Furthermore, the lipid droplet marker, PLIN2 and the sphingosine modifier, SPHK1, showed higher expression levels in the high-risk than the low-risk PDX. **d** Glucose consumption and lactate production showed highest levels in the high-risk PDX. Concomitantly, the glutamine metabolism was higher in the high-risk PDX. Error bars indicate mean ± SD. *P < 0.05, **P < 0.01.

Previous reports have demonstrated a strong association between metabolism and PDAC subtypes, highlighted by progenitor tumors displaying an energetic dependency on fatty acids (FA) and cholesterol^28,29^. In contrast, in high-risk patients the most undifferentiated ones upregulate glycolytic pathways^30^. These observations aligned with our transcriptomic and methylome characterization of PDAC patients following the MR-Gradient. Furthermore, there is an intricate link between the epigenome and metabolism with epigenetic changes impacting the transcription of metabolic genes to affect cell metabolism and central metabolites from diverse pathways serving as essential cofactors for chromatin-modifying enzymes^31–33^. Thus, we analyzed how metabolic networks are represented within the context of our MR-Gradient and its relationship to prognosis. We built a metabolic map using our PDX expression and methylome profiles of genes encoding key enzymes within central metabolic pathways (Supplementary Fig. 4; Supplementary Table 4). We found that enzymes such as ACSS1 (*r* = 0.61; *P* < 0.0001), ACACB (*r* = 0.45; *P* < 0.0001), and HMGCR (*r* = 0.49; *P* < 0.0001), which are involved in acetate metabolism, FA synthesis, and cholesterol anabolism, respectively, positively correlate with the MR-Gradient. This pro-lipidic metabolism was reflected by high expression of enzymes implicated in complex lipid biosynthesis from the glycerophospholipids and sphingolipids pathways (Supplementary Fig. 4; Supplementary Table 4). Conversely, the unfavorable phenotype augmented OXPHOS independent metabolism centered on the Warburg effect and glutaminolysis, where both amino acid synthesis and accumulation of triglycerides (TG) in lipid droplets play a central role (Supplementary Fig. 4). These observations were confirmed through lipidomic analysis of 72 PDXs, where 28 subfamilies were detected; however, only 6 displayed a significant association with the MR-Gradient (Fig. 7a; Supplementary Table 4). Specifically, the phosphoglyceroethanolamines (PE; *r* = 0.36; *P* = 0.002) showed positive correlation with the MR-Gradient, while the TG (*r* = −0.26; *P* = 0.03) and ceramides metabolites, such as Monohexosylceramides (CMH; *r* = −0.26; *P* = 0.02) and Sphingomyelin (SM; *r* = −0.26; *P* = 0.02), associated with a poor prognosis. Cox regression analysis following PCA of highly correlated metabolites of the selected subfamilies (uHR = 0.9, 95% CI [1.01–1.18]; *P* = 0.03; Fig. 7b) revealed a strong association between the TG and the ceramides metabolites with prognosis, constituting 53.84% and 24.6%, respectively, of the Dimension 1 contribution. In addition, SPHK1 and PLIN2 expression were assessed in PDXs to validate these proteins as markers of poor prognosis associated with sphingolipid metabolism and TG accumulation, respectively. Notably, both SPHK1 and PLIN2 display a higher number of positive cells in high-risk (76%–85%) compared with their representation in the low-risk (2%–12%) patients, which also positively correlate with the EMT marker vimentin (Fig. 7c). Lastly, we demonstrate that high-risk patients show a dependence on anaerobic glycolysis and glutaminolysis, by measuring their corresponding metabolites in supernatants from PDX explants. Glucose consumption and lactate production were 1.4 and 2.6 times higher, respectively, in high-risk patients *vs*. the low-risk group (Fig. 7d). Consistently, glutamine consumption and glutamate production increased in high-risk samples by 0.4 and 0.7 times, respectively (Fig. 7d). These results highlight ATP source and lipid metabolism as determinants of prognosis-related phenotypes.

## Discussion

The current study makes novel contributions to the field of PDAC that have both significant mechanistic and biomedical relevance. This study developed from the observation that a binary classification of PDAC into basal-like and classical subtypes does not completely capture the full heterogeneity of PDAC, nor it helps to stratify patients accurately, nor does it consider the contribution of the microenvironment. Consequently, the current PDAC molecular stratification analysis relies on transcriptomic/genomic data only, without integrating or characterizing other PDAC biological features, such as epigenetic or metabolic profiles. This limitation is accentuated by the tumor-centric focus of most classification and the lack of actionable clinical tools to differentially treat subtypes. Here, we provide a transcriptional regulator-centered model that allocates the patients into a prognosis continuum, incorporating both the tumoral cell and microenvironment compartments. We show that this model: (1) increases the power of diagnostic prediction; (2) allows to infer the major transcriptional networks that contribute to PDAC heterogeneity; (3) integration of the MR-Gradient model with epigenomics and metabolomics profiles improves diagnostic schemes and provides information on PDAC pathobiological, genome-wide pathways, and potential therapeutic targets; (4) identifies a key role for H3K9-mediated pathways (methylation vs acetylation) in PDAC; and (5) underscores the distinct association of lipid and glucose/OXPHOS metabolism with patients’ outcomes.

PDAC tumors are composed of two well-known strati, the tumor cell, and the microenvironment, where the interplay between those determines the global phenotypic, drug resistance, and invasiveness^4,6,34–36^. Despite the complexity and diversity of the manifest biology by PDAC tumors, the principles that underlay their behavior is common to any tissue, where the observed phenotype is determined through the action of a core of TFs which modulate the transcriptional network of the compartments previously mentioned. Thus, to understand and fully characterize the PDAC, we can refer to a selected group of proteins with a key role in defining the tumoral outcome. Initially, we isolated the tumoral prognosis determinants components applying LRICA. PAMG component was identified as a key driver of tumoral phenotype, confirming the biological relevance of gradient stratification^12^. However, the immunological component, extracted from the microenvironment compartment, displayed a significant contribution to the prognosis estimation, working as a correction factor of the tumor cell phenotype. We observed a positive correlation between a high immunological score and a good prognosis. Specifically, T cells and B cells were identified as strong conditional of PDAC prognosis. In fact, CD8A and CD27 markers showed the highest correlation with the immunological component, suggesting a role of cytotoxic T cells and antibody-producing B cells to improve the patient’s outcome. Similar results regarding T cells were detailed by Carstens et al.^37^ which used multiplex immunohistochemistry on 132 patients, demonstrating the survival improvement in high T cell PDAC infiltrating tumors. Interestingly, in contrast to our observations, CD20^+^ B cells have been proposed as detrimental for a proper antitumoral response^38^. This difference may be related to the types of B cell subpopulations identified, whereas CD20 is a pleiotropic marker, CD27 is circumscribed to an effector population. In fact our observation in PDAC better correlates with an active B cell anti-tumor response, such as observed in ovarian and lung cancer^39,40^. Consequently, to build our MR-Gradient we applied transcriptional network analysis on the prognosis-significant components, isolating the TFs that modulate the PDAC outcome. As expected PAMG component was defined mainly by the classical TFs, such as GATA6, HNF4A and ZFPM1 and secondary by poor prognosis factors, such as, SNAI2, and HMGA2, MYBL1, which are EMT and cell cycle modulators^14,17,22,23^. This regulatory profile between a pancreas lineage TFs and inducible cell processes TFs, reaffirm the gradient stratification approach, suggesting PDAC transitional states triggered by the poor prognosis TFs expression. The immunological TFs also can define both an inflammatory and a regulatory core, with proteins related with B/T cells and Treg, respectively. Surprisingly, the regulatory core displayed a positive correlation with the patient’s outcome, which is aligned with Zhang et al.^41^ observations, where the Treg depletion promotes the tumor progression through CD4^+^/myeloid cell immune-suppressive function and the myCAF depletion. Indeed, the enrichment in iCAF^6^ and MOFFITT_ACTIVATED_STROMA^8^ signatures associated with a MR-Gradient low score may be explained by loss of Treg induction within the PDAC microenvironment.

To extend this prognosis-mechanistic relationship through inferences made at the level of transcriptional regulation, we used multi-OMICs data derived from PDXs. Initially, we measured the divergence between the PDXs and the human tumor following our gradient model. As expected, a high recapitulation level was observed, both at epithelial tumor cell level as the microenvironment, confirming previous observations and validating their use to extract PDAC biological relevant information. Then, we correlated the methylation levels and histone code regulators expression with our MR-Gradient model. This analysis led us to identify a hypermethylation profile associated with a worse prognosis, specifically on the loci of favorable prognosis TFs and lipid metabolism proteins. Concomitantly, SUV39H1/2 and KAT2B, two histone writers with antagonist effects on H3K9 residue, were detected as PDAC phenotype determinants. Interestingly, while SUV39H1 and SUV39H2 have not been previously associated to PDAC initiation, progression, or prognosis, this mark is read by the HP1 family of proteins. Moreover, HP1 form a complex with DNA methyl transferases KAT2B, is critical for regulating a particular type of enhancers marked by H3K9ac, which differentiate them from the H3K27ac, a mark written by the CBP/P300 family of proteins^23,42^. KAT2B works with many transcription factors, among them many of the ones found in our MR networks^23^. Noteworthy, we found that genomic deletion of KAT2B occurs in 25% of our PDX samples and tumors from TCGA. KAT2B promoter methylation and downregulation also correlated to distinct prognosis and functional phenotypes. These observations led to experimental validations, which showed the cell nuclei from high-risk patients have a higher positive index for nuclear H3K9me3 mark. In contrast, a high positive index for the H3K9ac mark links to more favorable outcomes. Orthogonal confirmation of these observations was obtained using both pharmacological and genetic inhibition of these writers, resulting in the molecular changes congruent with their phenotype component. Therefore, our MR-Gradient is rich in new information regarding key molecular players and associated factors with a better-defined prognosis for PDAC.

Integration with metabolomic data indicates that acetate metabolism, FA synthesis, and cholesterol anabolism, positively correlate with the MR-Gradient. Conversely, the unfavorable phenotype augmented OXPHOS independent metabolism centered on the Warburg effect and glutaminolysis. These observations were confirmed through lipidomic analysis of 72 PDXs, which identified six pathways positively correlated with our MR-Gradient model. High-risk patients show a dependence on anaerobic glycolysis and glutaminolysis, which we confirmed by measuring their corresponding metabolites in supernatants from PDX explants. Glucose consumption and lactate production were higher in high-risk vs. low-risk group patients. Similarly, glutamine consumption and glutamate production were appropriately coupled. Moreover, the lipidomic analysis unravels a strong association between triacylglycerol accumulation and sphingosine metabolism with the high-risk phenotype, which was confirmed using PLIN2 and SPHK1, respectively, which have been described as determinants of patients’ outcome in other types of cancers^43,44^. These results highlight ATP source and lipid metabolism as determinants of prognosis-related phenotypes. These observations are important in light that these mechanisms have been previously described to play a role in pancreatic cancer in both animal models and humans, though their relationship to a distinct prognosis scheme had not been stringently established. Furthermore, their relationship with transcriptional regulation and epigenomics remains an unfilled paradigm.

In summary, the current study offers a robust integration for valuable predictions of patient prognosis through transcriptional networks, DNA methylation, epigenomic regulators, and metabolomics, mechanisms that bear prognostic and mechanistic value and uncover potential therapeutic targets to fight this disease.

## Methods

### Ethical approval

The study was approved by the local ethics committee (Comité de protection des personnes Sud Méditerranée I) following patient informed consent. The PaCaOmics study is registered at www.clinicaltrials.gov with registration number NCT01692873. Written consent forms of informed patients were collected and registered in a central database. PDAC samples were collected from January 2012 to December 2015. All experimental procedures on animals were approved by the Comité d’éthique de Marseille numéro 14 (C2EA-14).

### Derivation of patient-derived xenograft (PDX) and PDX-derived primary cell culture (PDPCC)

PDAC samples were obtained from three expert clinical centers under the PaCaOmics clinical trial (number 2011-A01439-32) after receiving ethics review board approval. Consent forms of informed patients were collected and registered in a central database. In addition, animal experiments were performed following the institutional guidelines and were approved by the “Plateforme de Stabulation et d’Expérimentation Animale” (PSEA, Scientific Park of Luminy, Marseille). Ninety PDXs were used in this study which were generated as previously described^4^. Briefly, PDAC tissue was fragmented and mixed with 100 μl of Matrigel and implanted subcutaneously in a NMRI-nude mouse until the tumor reached a 1 cm^3^ (Swiss Nude Mouse Crl: NU(lco)-Foxn1nu; Charles River Laboratories, Wilmington, MA). PDPCC were obtained from splitted PDXs into small pieces of 1 mm^3^ and dissociated with collagen type V C9263; Sigma-Aldrich, Inc., St. Louis, Missouri, USA) and trypsin/EDTA (25200‐056; Gibco, Sigma-Aldrich, Inc., St. Louis, Missouri, USA). Cell homogenate was resuspended in DMEM with 1% w/w penicillin/streptomycin (Gibco, Life Technologies) and 10% of fetal bovine serum (Lonza). After centrifugation, cells were re‐suspended in Serum Free Ductal Media (SFDM) adapted from Schreiber et al.^45^ and conserved at 37 °C in a 5% CO_2_ incubator. Both, PDX and PDPCC RNA, was isolated with the miRneasy mini kit (Qiagen). RNA-seq reads were mapped using STAR. Gene expression profiles were obtained using Feature Count and normalized using Trimmed Mean of M-values approach. In addition, SMAP algorithm^4^ was applied to separate human and mice reads from PDX RNA-Seq data.

### Low-rank independent component analysis (LRICA) and prognosis-related component extraction

To identify the intrinsically biological trend into the PDAC biology a modified version Low-Rank ICA (LRICA) was applied^19^. Initially we integrated three RNA-Seq expression datasets, TCGA-PAAD, ICGC-PACA-AU Seq, and PDX previous common normalization and log2 transformation. TCGA-PAAD, and ICGC-PACA-AU Seq were curated using previously described criteria^7^. Robust PCA^46^ was used to decompose the integrated matrix into the low rank matrix (L) and the sparse matrix (S), capturing the constitutive dynamics and de noise of the original dataset, respectively. The next goal was to isolate the major biological trends transversal to the PDAC phenotype, independently of the cohort or sequency platform. Thus, we deconvoluted the L matrix into independent factors with the ProDenICA algorithm from the ProDenICA R package^47^, following the iterative process described previously^48^ to confirm the reproducibility of the selected components. The ICA deconvolution results in an W and S matrices, representing the sample orthonormal matrix and the gene contribution matrix for each component, respectively. Then, we weight the biological relevance and directionality of each component on the S matrix, calculating the kurtosis excess and gene-set enrichment analysis (GSEA) using e1071 and fgsea R packages, respectively. Components with a high kurtosis and enriched in a set of pathways associated biologically were selected. After that, further component discrimination following the prognosis criteria was done through Kaplan–Meier (KM) analysis on the W matrix. The group stratification per component was established through optimal *P* value separation. Finally, component weight on prognosis estimation was determined applying Principal Component Analysis (PCA) on KM analysis selected components, followed of Cox proportional hazard model on the PCA sample dimension coordinates. The dimension with the lowest Cox model *P* value was extracted and used for downstream analysis.

### Inference of stroma cell type abundance from bulk RNA and their prognosis association

We applied MCP-counter algorithm^49^ to estimate sample’ immune infiltration and stroma cell abundance of the transcriptomic data from TCGA-PAAD and ICGC-PACA-AU Seq. Briefly, MCP-counter cell type identification process relies on transcriptomic markers with a high cell type specificity. Consequently, the log_2_ geometric mean of the set of markers for each cell category is computed and used as the abundance score. Spearman’s correlation was applied to determine the association between the MCP-counter scores and the PCA-selected dimension. Cell type with an *r* > 0.2 and a *P* < 0.05 were identified as strongly related with the prognosis. In addition, a subset of specific lymphocyte T and B markers were used to confirm the cell subtype specificity.

### Master regulators (MR) gradient generation and validation

To build a robust PDAC stratification system using bulk RNA we used the core of transcriptional factors (TFs) for each prognosis-relevant component as phenotype drivers, weighting their contribution into the global score. Initially, we identified the highly contributive TFs into each selected component through Gene Ontology (GO) annotation (GO:0001067; Regulatory region nucleic acid binding). A total of 113 TFs were extracted, representing the PAMG, Immunological, and Stroma, components. Then, the regulatory network was inferred for each TF using the RTN R package^21^. The regulons (set of genes regulates by a TF) were detected and depurated with the ARACNe algorithm to eliminate redundant associations^20^. This list of regulons supported the Master Regulator (MR) Analysis applying as targets the set of genes with a high contribution (>2 SD) into each component. 78 MR were detected with a *P* < 0.01 (Supplementary Table 1). Once identified, we established the weight of each MR into the global phenotype using GSEA-2T, which calculate two enrichment score according with the genes positively and negatively regulates into each regulon following the sample distribution into the component. The differences between these two enrichment scores indicates activation or repression of a particular regulon and constitutes the MR weight factor. The MRs with an absolute dES ≥ 1 were chosen to build the MR-Gradient following the Eq (1):

$${{{\boldsymbol{Gradient}}}} = {{{\boldsymbol{Scale}}}}\left[ {\mathop {\sum}\limits_{\begin{array}{*{20}{c}} {{{{\boldsymbol{i}}}} = {{{\bf{1}}}}} \\ {{{{\boldsymbol{J}}}} = {{{\bf{1}}}}} \end{array}}^\infty {\left( {{{{\boldsymbol{ES}}}}_{{{{\boldsymbol{iJ}}}}} \times {{{\boldsymbol{GE}}}}_{{{{\boldsymbol{iJ}}}}}} \right)} } \right]$$

where each gradient is computed, weighting the gene expression (GE) with the enrichment score (ES) for each MR (i) and patient (j), followed by the scaled summation. Each component gradient and their mix were computed to identify the combinatory set of biological components with the lowest *P* value into the Cox Proportional Hazard model. The MR-Gradient estimation and reliability was validated in Puleo cohort^5^ and ICGC-PACA-AU Array.

### MR-Gradient transcriptomic phenotype characterization

To extract the specific phenotype that explain the MR-Gradient model we applied independent component analysis (ICA) on the integrated TCGA-PAAD and ICGC-PACA-AU Seq matrix using the JADE algorithm in MineICA R package^50^. Spearman’ correlation was used to identify the component with the higher/significant association with the estimated MR-Gradient model.

### Analysis of patient-derived xenograft (PDX) associated with MR-gradient stratification

Initially, differential expression analysis was performed using Limma R package on the PDX’ epithelial tumoral cell (human) and stroma matrices, comparing the specific ortholog genes to estimate the enrichment in the MR-Gradient transcriptional factors. Then, we added the PDX’ stroma expression matrix (mice) with the epithelial tumoral cell matrix (human) to calculate the MR-Gradient model. ICA JADE algorithm from the MineICA R package together with spearman’ correlation was applied to extract the PDX phenotype related with the MR-Gradient. After that, we correlated the ICA components from the integrated TCGA-PAAD/ICGC-PACA-AU Seq matrix and the PDX matrix to evaluate the degree of recapitulation of PDX model. This was performed on the global gene set and the subset of specific genes from the tumoral epithelial cell and the stroma using log2 fold change ≥ 1 and a false discovery rate (FDR) < 0.05.

### DNA methylation analysis

PDX whole-genome DNA methylation was analyzed as previously described^23^. Briefly, microarray experiments and hybridized to the BeadChip arrays were carried out at Integragen SA (Evry, France) following the manufacturer’s instructions. Illumina GenomeStudio software was used to extract the probe DNA methylation intensity signal values for each locus. Data were processed and normalizing following the workflow detailed in the methylationArrayAnalysis R package. In addition, we used ICA to capture the methylation profile associated with the CGM applying the JADE algorithm and Spearman’s correlation. The methylation level associated with the gradient was measured through the median of the CpG set with a component absolute contribution ≥ 3 SD. CpGs gene-set enrichment analysis (GSEA) was performed using the missMethyl R package.

### Inhibition of SUV39H1/2 on PDPCC and RNA-seq analysis

Two hundred thousand cells were seeded on a T25 flask in SFDM. Twenty-four hours later the media was supplemented with a sublethal concentration of Chaetocin (10 nM; Selleckchem, Houston, Texas, USA) and incubated for 72 h. Control samples were treated with DMSO only. After that, RNA was extracted using RNeasy mini kit (Qiagen). RNA libraries were prepared (Illumina NextSeq 500 High output kit v2) and run on the Illumina NextSeq for 75 bp paired end reads. Expression matrix were obtained using Rsubread R package^51^. Differential expression analysis was performed with the Limma R package. In addition, principal component analysis (PCA) was computed on the genes with a log2 fold change ≥ 1 and a false discovery rate (FDR) < 0.05. The distance between control and treated PDPCC was computed accounting the coordinates from the dimension with the higher explained variance.

### KAT2B siRNA transfection and RNA-seq analysis

Four KAT2B siRNA (L-005055-00-0010, ON-TARGETplus siRNA Reagents, Dharmacon) were transfected in two hundred thousand cells seeded on a six-well plate using INTERFERin reagent (Polyplus-transfection) according to the manufacturer’s protocol. The sequences of KAT2B-specific siRNAs were as follows: KAT2B-1: 5′-GGUACUACGUGUCUAAGAA-3′; KAT2B-2: 5′-GAGCCGACCUGCAGCAAAU-3′; KAT2B-3: 5′-CGACAGAUUCCUAUAGAAA-3′; and KAT2B-4: 5′-GCAAACAAUAGUUGAGUUG-3′. A control siRNA pool was used as the negative control (D-001810-10-05, ON-TARGETplus siRNA Reagents, Dharmacon). After 72 h, cells were lysed, and RNA extracted with RNeasy Mini Kit (Qiagen). RNA libraries were prepared (Illumina NextSeq 500 High output kit v2) and run on the Illumina NextSeq for 75 bp paired end reads. Differential expression analysis was performed with the Limma R package. In addition, principal component analysis (PCA) was computed on the genes with a log2 fold change ≥ 1 and a false discovery rate (FDR) < 0.05. The distance between control and treated PDPCC was computed accounting the coordinates from the dimension with the higher explained variance.

### Functional analysis

To characterize the pathways related to the selected ICA component and differential expression analysis, a gene-set enrichment analysis (GSEA) was performed using fgsea R package, which implements GSEA on a pre-ranked list of genes and MsigDB signaling database.

### CNV of PDX analysis

The copy number status of the PDX KAT2B was assessed using Illumina Infinium HumanCode-24 BeadChip SNP at Integragen SA (Evry, France), according to the manufacturer’s recommendations. The BeadStudio software (Illumina) was used to normalize raw fluorescent signals and to obtain log R ratio (LRR) and B allele frequency (BAF) values. Asymmetry in BAF signals due to bias between the two dyes used in Illumina assays was corrected using the tQN normalization procedure.

### Protein extraction and Western Blot

The proteins were separated by SDS-PAGE (29:1 acrylamide:bis-acrylamide, Euromedex Laboratories, France) in 10%–12% running gel and 4% stacking gel, in an electrophoresis cell. Proteins were electro-transferred to a nitrocellulose membrane (Immobilon-P, EMD Millipore Corporation, Billerica, Massachusetts, USA) at 250 mA for 2 h. To identify proteins, the membranes were blocked for 1 h at room temperature with 5% powdered milk in PBS containing 0.1% Tween 20. Next, they were incubated overnight at 4 °C with the rabbit polyclonal antibodies anti-H3K9me3 (1:2000, C15410056, Diagenode), and anti-H3K9ac (1:1000, 9649, Cell Signaling Technology, USA). For the immunoreaction, the membranes were incubated with horseradish peroxidase (HRP)-conjugated goat anti-rabbit IgG (1:3000, 4030-05, Suther Biotech, Birmingham, USA). The outcome was visualized using the Chemiluminescent HRP substrates (Millipore Corporation, Burlington, Massachusetts, USA) for chemiluminescence development. To normalize the results, polyclonal anti-H3 (1:1000, 14269, Cell Signaling Technology, USA) was used on the same membranes. The membranes were scanned using a PXi multi-application imager (Sygene, Cambridge, UK). The estimation of bands was performed using a prestained protein ladder (SeeBlue Plus2, ThermoFisher, Waltham, Massachusetts, USA) as a molecular weight marker.

### Immunocytofluorescence and nuclear localization quantification

Cells were grown on commercial microscope slide glasses, fixed with PFA solution, washed twice with PBS and with blocking serum solution (Vector Laboratories, Burlingame, CA, USA) for 30 min. Then, the cells were incubated with the primary antibody overnight at 4 °C. The primary antibody used was rabbit anti-H3K9me3 (1:200, C15410056, Diagenode). The secondary antibody was Alexa 488-conjugated anti-rabbit (1:300, A21206, Invitrogen, ThermoFisher, Waltham, Massachusetts, USA), which was incubated with the sections for 60 min at room temperature. The slides were counterstained with mounting medium for fluorescence with DAPI (ProLong, Invitrogen, ThermoFisher, Waltham, Massachusetts, USA). One hundred cells were selected randomly to quantify the histone mark signal. Images were captured using the microscope (Axio Imager 2, Zeiss, Germany) with an attached digital camera (ORCA-Fusion; Hamamatsu, Japan).

### Immunofluorescence and signal quantification

PDX paraffin sections were dewaxed in xylene and hydrated through a decreasing ethanol series. After 10 min in PBS, heat-induced epitope retrieval was performed in a water bath at 96 °C in 10 mM sodium citrate at pH 6 for 20 min. Then, the sections were blocked with blocking serum solution for 30 min. Slides were incubated with the primary antibody overnight at 4 °C. The primary antibodies used were rabbit anti-H3K27ac (1:100, 8173, Cell Signaling Biotechnology), anti-H3K4me3 (1:100, ab8580, Abcam), anti-H3K9me3 (1:100, C15410056, Diagenode), anti-H3K9ac (1:200, Cell Signaling Biotechnology), PLIN2 (1:100, NB110-40877, Novus Biologicals), and anti-SPHK1 (1:100, H00008877-M01, Novus Biologicals). The secondary antibody used was Alexa 488-conjugated anti-rabbit (1:300, A21206, Invitrogen, ThermoFisher, Waltham, Massachusetts, USA), which were incubated with the sections for 60 min at room temperature. The slides were counterstained with mounting medium for fluorescence with DAPI (ProLong, Invitrogen, ThermoFisher, Waltham, Massachusetts, USA). One hundred cells in three areas per section were used to quantify the signal. Images were captured using the microscope (Axio Imager 2, Zeiss, Germany) with an attached digital camera (ORCA-Fusion, Hamamatsu, Japan).

### Lipidomic analysis

Methanol and a mix of sodium chloride and chloroform/methanol (2:1) were used to isolate lipids from 77 PDX. Raw data were extracted using mass spectrometry coupled to ultra-performance liquid chromatography (UPLC-MS). Chromatography was performed using an ACQUITY™HPLC system (Waters Corp., Milford, USA), associated with the mass spectrometer Waters LCT Premier (Waters Corp., Milford, USA). All the measures included three defined quality control samples used to batch normalization. Raw data were processed using the TargetLynx application manager for MassLynx 4.1 software (Waters Corp., Milford, USA). A set of predefined retention time, mass-to-charge ratio pairs, Rt-*m/z*, corresponding to metabolites included in the analysis are considered. Associated extracted ion chromatograms (mass tolerance window = 0.05 Da) are then peak-detected and noise-reduced in both the LC and MS domains. A list of chromatographic peak areas is then generated for each sample injection. Normalization factors were calculated for each metabolite by dividing their intensities in each sample by the recorded intensity of an appropriate internal standard in that same sample, following the procedure described by Martinez-Arranz et al.^52^. A total of 28 subfamilies were detected and the median per sample was computed per each one. The lipid subfamilies with a significant statistical correlation with the MR-Gradient model were selected. Then, the metabolites into the selected subfamilies with a high spearman’s correlation with the MR-Gradient were analyzing with principal component analysis (PCA) to weight the metabolite contribution into the PDAC prognosis through the PCA dimension coordinates applied on Cox proportional hazard model.

### Glucose and glutamine metabolism

PDX explants of 1 mm^2^ extracted from three high-risk and three low-risk patients were seeding in a 12-well plate coated with 150 µl growth factor reduced Matrigel (Corning, Wiesbaden, Germany) per duplicate. Glucose and glutamine consumption together with lactate and glutamate production were measured using the YSI 2950 BioAnalyser (System-C-Industry). The explants were cultured in DMEM with 25 mM glucose, 2.5 mM glutamine. After 48 h, explant supernatants were collected to metabolites measurement. Raw data were normalized with the dry tissue weight.

### Statistical analysis

Spearman’s correlation coefficients and the significance levels were calculated using the Hmist R package. Mann–Whitney’s test, and Chi-Square test were performed with R basic functions. Heatmap and correlograms were generated with ComplexHeatmap R package.

### Reporting summary

Further information on research design is available in the Nature Research Reporting Summary linked to this article.

## Supplementary information

REPORTING SUMMARY

Supplementary Figures

Supplementary Table 1

Supplementary Table 2

Supplementary Table 3

Supplementary Table 4

## Data Availability

ICGC-PACA-AU Seq and ICGC-PACA-AU Array expression datasets were downloaded from the ICGC data portal (https://dcc.icgc.org/). TCGA-PAAD RNA expression and methylation data were downloaded with TCGAbiolinks R package. Puleo cohort data is available in ArrayExpress under the accession number E-MTAB-6134. PDX datasets are available from ArrayExpress and European Genome-phenome Archive under the accession numbers: E-MTAB-6134, E-MTAB-5039, E-MTAB-5008, E-MTAB-5006, and EGAS00001001928. TCGA-PAAD genomic data was extracted from cBioPortal (https://www.cbioportal.org/).

## Code availability

Data analysis was performed R (3.6.3) and RStudio (Version 1.1.453) with the packages described in the methods. Raw code will be made available from the corresponding author upon reasonable request.
