# Supplementary material for: Multi-omics data integration and modeling unravels new mechanisms for pancreatic cancer and improves prognostic prediction
Source: NPJ Precis Oncol. 2022 Aug 17;6:57. doi: 10.1038/s41698-022-00299-z (PMC9385633; doi:10.1038/s41698-022-00299-z)
Supplement: Supplementary file 1 — REPORTING SUMMARY [file 41698_2022_299_MOESM1_ESM.pdf]

nature portfolio

Corresponding author(s): Juan Iovanna

Last updated by author(s): Apr 29, 2022

Reporting Summary

Nature Portfolio wishes to improve the reproducibility of the work that we publish. This form provides structure for consistency and transparency in reporting. For further information on Nature Portfolio policies, see our Editorial Policies and the Editorial Policy Checklist.

Statistics

For all statistical analyses, confirm that the following items are present in the figure legend, table legend, main text, or Methods section.

n/a	Confirmed	
☐	☒	The exact sample size (*n*) for each experimental group/condition, given as a discrete number and unit of measurement
☐	☒	A statement on whether measurements were taken from distinct samples or whether the same sample was measured repeatedly
☐	☒	The statistical test(s) used AND whether they are one- or two-sided<br>*Only common tests should be described solely by name; describe more complex techniques in the Methods section.*
☐	☒	A description of all covariates tested
☐	☒	A description of any assumptions or corrections, such as tests of normality and adjustment for multiple comparisons
☐	☒	A full description of the statistical parameters including central tendency (e.g. means) or other basic estimates (e.g. regression coefficient) AND variation (e.g. standard deviation) or associated estimates of uncertainty (e.g. confidence intervals)
☐	☒	For null hypothesis testing, the test statistic (e.g. *F*, *t*, *r*) with confidence intervals, effect sizes, degrees of freedom and *P* value noted<br>*Give P values as exact values whenever suitable.*
☒	☐	For Bayesian analysis, information on the choice of priors and Markov chain Monte Carlo settings
☒	☐	For hierarchical and complex designs, identification of the appropriate level for tests and full reporting of outcomes
☒	☐	Estimates of effect sizes (e.g. Cohen's *d*, Pearson's *r*), indicating how they were calculated

Our web collection on statistics for biologists contains articles on many of the points above.

Software and code

Policy information about availability of computer code

Data collection	-PDX DNA methylation microarray experiments was carried out at Integragen SA (Evry, France) following the manufacturer's instructions.<br>-PDX lipidomic raw data was extracted using mass spectrometry coupled to ultra-performance liquid chromatography (UPLC-MS). Chromatography was performed using an ACQUITY™HPLC system (Waters Corp., Milford, USA), associated with the mass spectrometer Waters LCT Premier (Waters Corp., Milford, USA).<br>-Glucose and glutamine consumption together with lactate and glutamate production were measured using the YSI 2950 BioAnalyser (System-C-Industry).<br>-Images were captured using the microscope (Axio Imager 2; Zeiss, Germany) with an attached digital camera (ORCA-Fusion; Hamamatsu, Japan).<br>-Western blot membranes were scanned using a PXi multi-application imager (Sygene, Cambridge, UK).<br>-KAT2B siRNA and Chaetocin RNA-Seq were produced by Illumina NextSeq<br>-ICGC-PACA-AU Seq and ICGC-PACA-AU Array expression datasets were downloaded from the ICGC data portal (https://dcc.icgc.org/). TCGA-PAAD was downloaded with TCGAbiolinks R package. Puleo cohort and PDX datasets were downloaded from ArrayExpress (https://www.ebi.ac.uk/arrayexpress/) under the accession numbers: E-MTAB-6134, E-MTAB-5039, and E-MTAB-5008. TCGA-PAAD genomic data was extracted from cBioPortal.
Data analysis	All data was analyzed using open source R packages, with detailed descriptions in the Methods section.

For manuscripts utilizing custom algorithms or software that are central to the research but not yet described in published literature, software must be made available to editors and reviewers. We strongly encourage code deposition in a community repository (e.g. GitHub). See the Nature Portfolio guidelines for submitting code & software for further information.

Data

Policy information about availability of data

All manuscripts must include a data availability statement. This statement should provide the following information, where applicable:

- Accession codes, unique identifiers, or web links for publicly available datasets
- A description of any restrictions on data availability
- For clinical datasets or third party data, please ensure that the statement adheres to our policy

AII URL's and accession codes for the transcriptomic, methylomic, and genomic data that were used in this study are provided in the Data Availability Statement of the text. Lipidomic and KAT2B siRNA and Chaetocin RNA-Seq are available from the corresponding author on reasonable request.

Field-specific reporting

Please select the one below that is the best fit for your research. If you are not sure, read the appropriate sections before making your selection.

☒ Life sciences ☐ Behavioural & social sciences ☐ Ecological, evolutionary & environmental sciences

For a reference copy of the document with all sections, see nature.com/documents/nr-reporting-summary-flat.pdf

Life sciences study design

All studies must disclose on these points even when the disclosure is negative.

Sample size	No statistical methods were used to determine the sample size. For the transcriptomic, methylomic, genomic and metabolomic analysis, we used the complete PDX datasets. For the publicly available datasets, ICGC-PACA-AU Seq, ICGC-PACA-AU, Puleo, and TCGA-PAAD, we used the primary tumors with confirmed PDAC diagnosis.
Data exclusions	No data were excluded from the analyses.
Replication	For in vitro experiments, at least three biological replicates were performed with technical replicates per experiment whenever feasible.
Randomization	This is descriptive research, so no allocation and randomization was used.
Blinding	This is descriptive research, so no blinding was used.

Reporting for specific materials, systems and methods

We require information from authors about some types of materials, experimental systems and methods used in many studies. Here, indicate whether each material, system or method listed is relevant to your study. If you are not sure if a list item applies to your research, read the appropriate section before selecting a response.

Materials & experimental systems

n/a	Involved in the study	
☐	☒	Antibodies
☐	☒	Eukaryotic cell lines
☒	☐	Palaeontology and archaeology
☐	☒	Animals and other organisms
☒	☐	Human research participants
☒	☐	Clinical data
☒	☐	Dual use research of concern

Methods

n/a	Involved in the study	
☒	☐	ChIP-seq
☒	☐	Flow cytometry
☒	☐	MRI-based neuroimaging

Antibodies

Antibodies used	Primary antibodies for anti-H3K9me3 (Diagenode), and anti-H3K9ac (Cell Signaling Technology, USA), anti-H3 (Cell Signaling Technology, USA), anti-H3K27ac (Cell Signaling Biotechnology), anti-H3K4me3 (Cell Signaling Biotechnology), PLIN2 (Novus Biologicals), and anti-SPHK1 (Novus Biologicals). Secondary antibodies horseradish peroxidase (HRP)-conjugated goat anti-rabbit IgG (Suther Biotech, Birmingham, USA), Alexa 488-conjugated anti-rabbit (Invitrogen, ThermoFisher).
Validation	All the antibodies are commercially available and have been validated by the manufacturer

Eukaryotic cell lines

Policy information about cell lines

Cell line source(s)	All the cell lines used in this study were derived in our laboratory from PDX.
Authentication	All the cell were autenticated by KRAS mutation
Mycoplasma contamination	All cell lines were tested for Mycoplasma at regular intervals using MycoAlert Mycoplasma Detection kit (Lonza)
Commonly misidentified lines (See ICLAC register)	Commonly misidentified cell lines were not used.

Animals and other organisms

Policy information about studies involving animals; ARRIVE guidelines recommended for reporting animal research

Laboratory animals	NMRI-nude mouse (Swiss Nude Mouse Crl: NU(lco)-Foxn1nu; Charles River Laboratories, Wilmington, MA)
Wild animals	The study did not involve wild animals
Field-collected samples	The study did not involve samples collected from the field
Ethics oversight	All animal experiments were conducted in accordance with institutional guidelines and were approved by the “Plateforme de Stabulation et d’Expérimentation Animale” (PSEA, Scientific Park of Luminy, Marseille)

Note that full information on the approval of the study protocol must also be provided in the manuscript.
