# Supplementary material for: Multi-omics data integration and modeling unravels new mechanisms for pancreatic cancer and improves prognostic prediction
Source: NPJ Precis Oncol. 2022 Aug 17;6:57. doi: 10.1038/s41698-022-00299-z (PMC9385633; doi:10.1038/s41698-022-00299-z)
Supplement: Supplementary file 2 — Supplementary Figures [file 41698_2022_299_MOESM2_ESM.pdf]

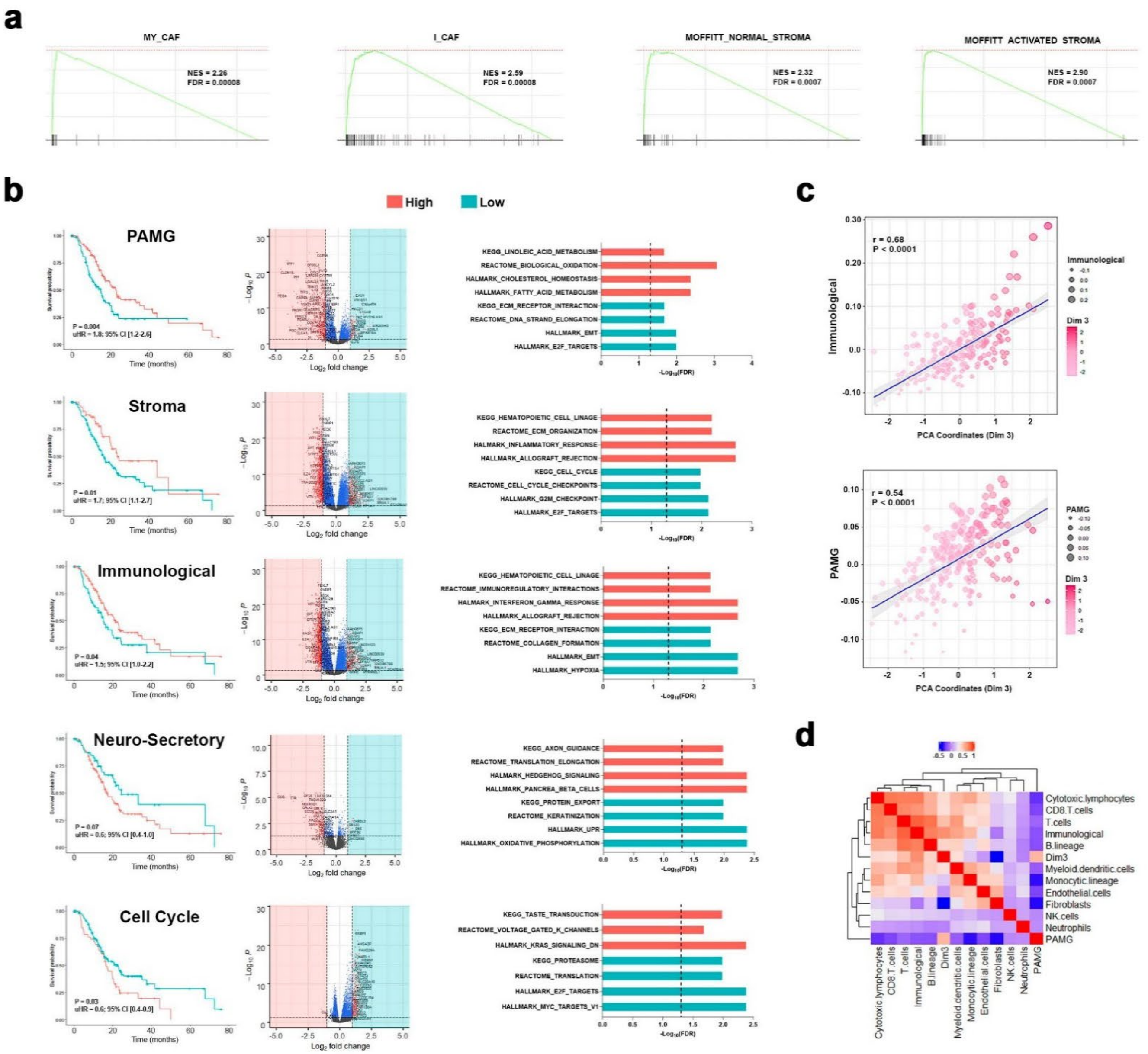

Supplementary Figure 1. Characterization of selected components. a) GSEA of the stroma component associated with key microenvironment prognosis related signatures. **b**) Kaplan-Meier analysis and Differential expression analysis of the biological relevant components together with the GSEA. **c**) Dimension 4 (Dim 4) association with the immunological and PAMG component. **d**) Correlogram of the MCP-Scores with the PCA dimension 3 (Dim 3) coordinates, PAMG and immunological components.

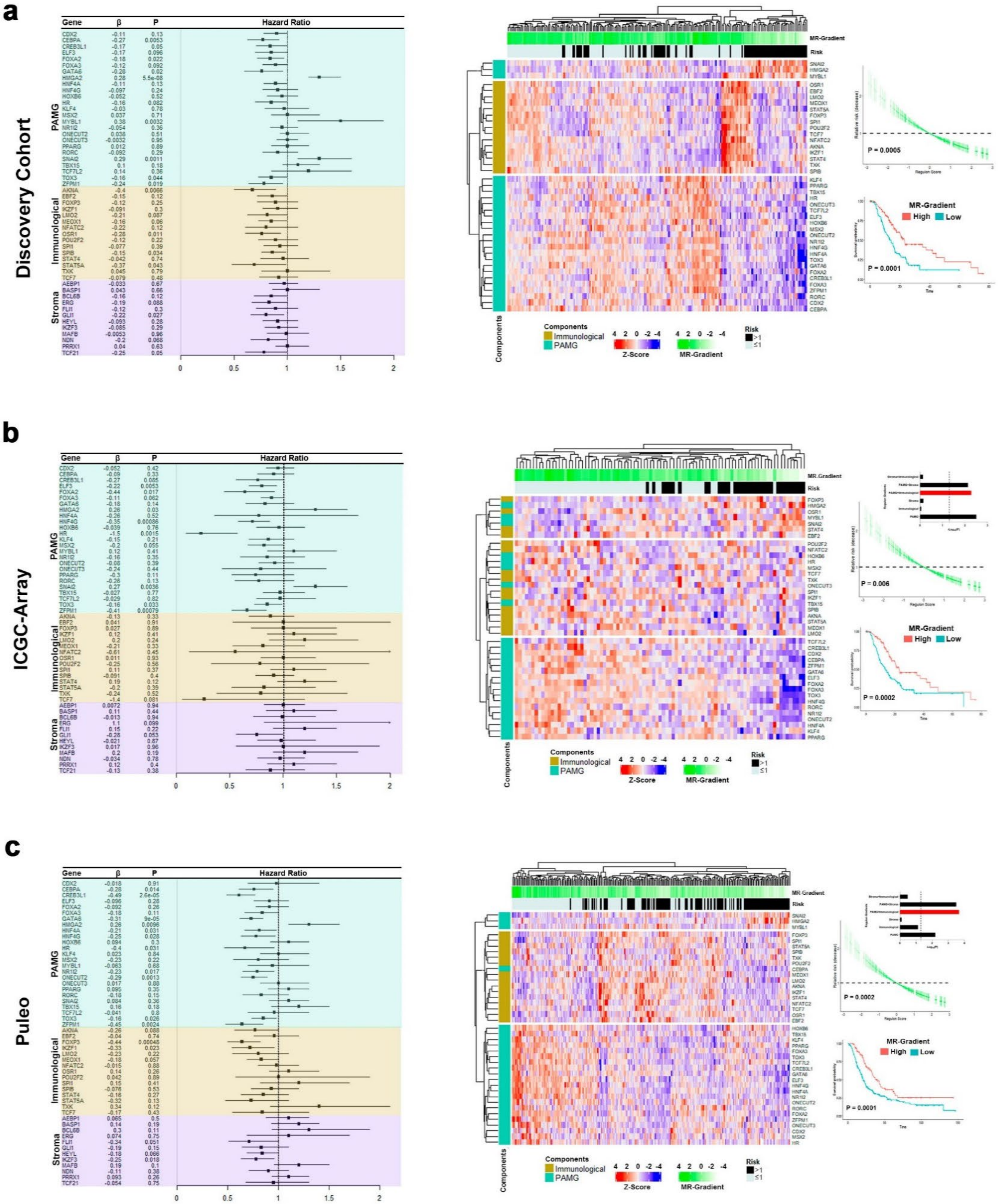

Supplementary Figure 2. Validation of MR Gradient in PDAC human cohorts. Univariate Cox regression analysis of the master regulator (MR) and expression profile of MR, Cox regression and Kaplan-Meier analyses of the MR-gradient was performed on the **a**) discovery cohort, **b**) ICGC-Array cohort, and **c**) Puleo cohort.

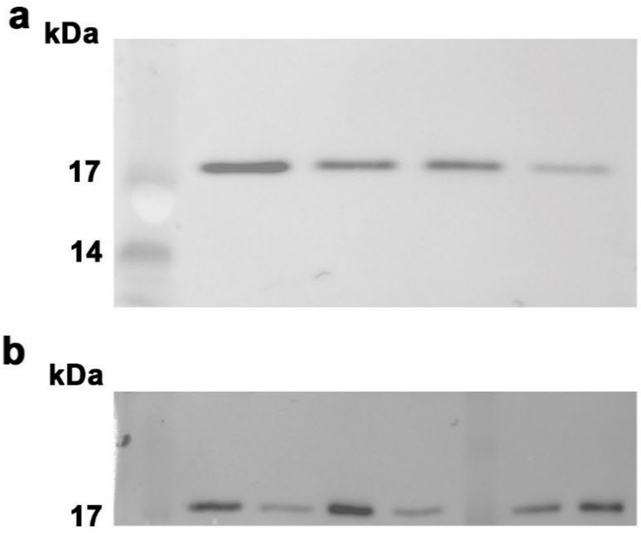

Supplementary Figure 3. Expression levels of histone modifications in treated PDAC cells. a) Western blot H3K9me3. **b**) Western blot H3K9ac.

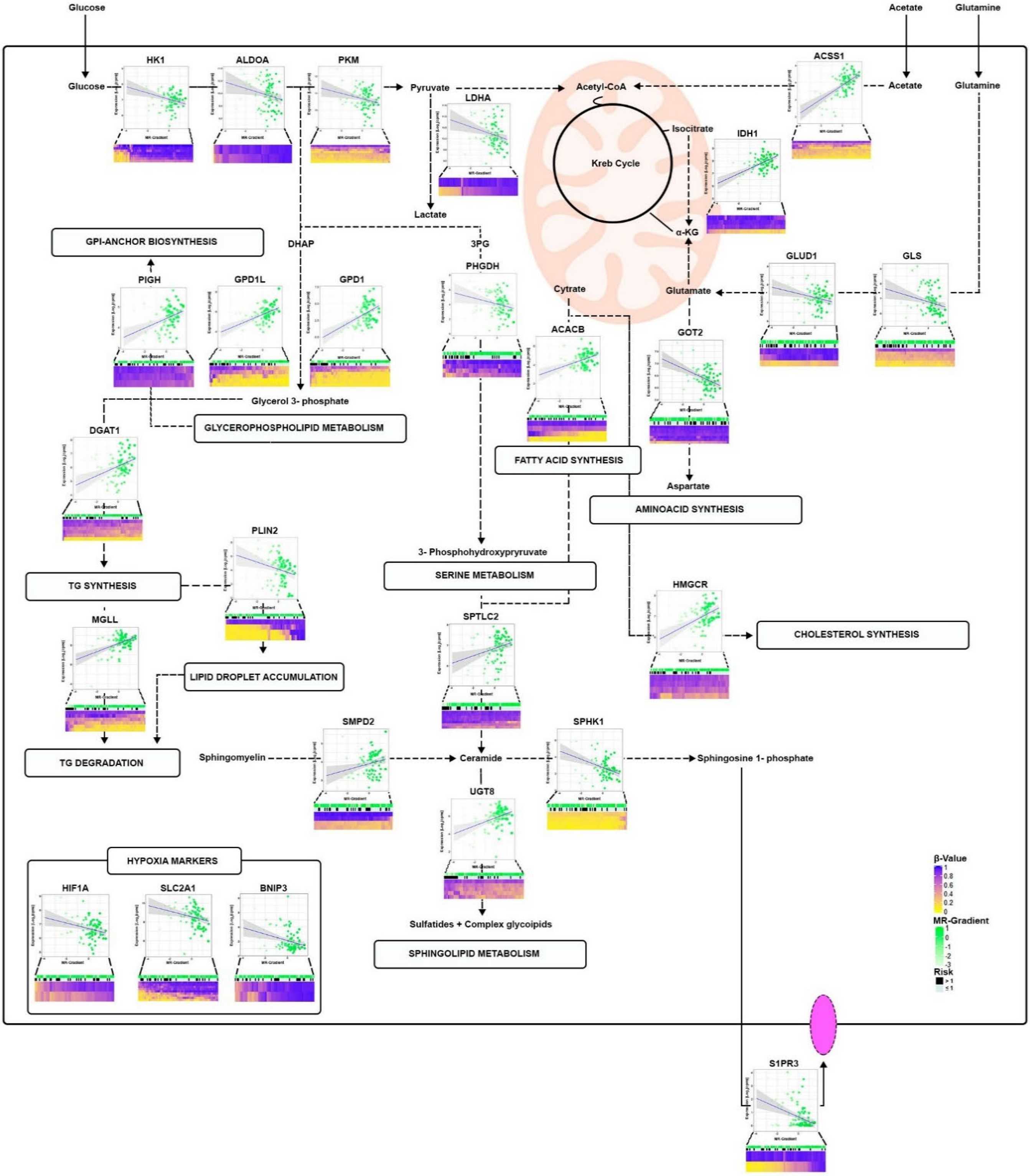

Supplementary Figure 3. PDAC metabolic map following the MR-Gradient.

Supplemental Tables:

Table 1. Data used to extract the PDAC biological relevant components and the MR-Gradient.

Table 2. Data from ICA to evaluate the recapitulation level of PDX.

Table 3. Data derived from the association between the epigenetic and MR-Gradient.

Table 4. Data extracted from the PDAC metabolic profile related with the MR-Gradient.
